# Supplementary material for: WWOX Controls Cell Survival, Immune Response and Disease Progression by pY33 to pS14 Transition to Alternate Signaling Partners
Source: Cells. 2022 Jul 7;11(14):2137. doi: 10.3390/cells11142137 (PMC9323965; doi:10.3390/cells11142137)
Supplement: Supplementary file 1 [file cells-11-02137-s001.zip › Supplemental Video Legends.pdf]

Supplemental Video Legends

Video S1. Ceritinib induces individual breast 4T1 cell apoptosis and cell sphere explosion and death. 4T1 cells were pretreated with 10 µl non-immune rabbit serum for 30 min, followed by treating with 30 µM ceritinib for imaging the cell death event by time-lapse microscopy (15 min per frame). Note that the cells picked up DAPI first (without death; blue) and then PI (red) in dead cells. The stem cell sphere underwent explosion and then death. (Data from reference "Wang et al., 2019")

Video S2. $WWOX_{286-299}$ antiserum does not block ceritinib-induced death of breast 4T1 cells. Under similar conditions, 4T1 cells were pretreated with 10 µl WWOX antiserum against the $WWOX_{286-299}$ peptide for 30 min, followed by treating with 30 µM ceritinib for imaging of cell death by time-lapse microscopy (15 min per frame). (Data from reference "Wang et al., 2019")

Video S3. $WWOX_{7-21}$ antiserum blocked ceritinib-induced death of breast 4T1 cells. Under similar conditions, 4T1 cells were pretreated with 10 µl WWOX antiserum against $WWOX_{7-21}$ peptide for 30 min, followed by treating with 30 µM ceritinib for imaging of cell death by time-lapse microscopy (15 min per frame). (Data from reference "Wang et al., 2019")
